# An engineered bacterium auxotrophic for an unnatural amino acid: a novel biological containment system

**DOI:** 10.7717/peerj.1247

**Published:** 2015-09-15

**Authors:** Yusuke Kato

**Affiliations:** Genetically Modified Organism Research Center, National Institute of Agrobiological Sciences, Tsukuba, Ibaraki, Japan

**Keywords:** Biological containment, Colicin, Mutation rate, Toxin–antitoxin system, Unnatural amino acids, Auxotrophy

## Abstract

Biological containment is a genetic technique that programs dangerous organisms to grow only in the laboratory and to die in the natural environment. Auxotrophy for a substance not found in the natural environment is an ideal biological containment. Here, we constructed an *Escherichia coli* strain that cannot survive in the absence of the unnatural amino acid 3-iodo-_L_-tyrosine. This synthetic auxotrophy was achieved by conditional production of the antidote protein against the highly toxic enzyme colicin E3. An amber stop codon was inserted in the antidote gene. The translation of the antidote mRNA was controlled by a translational switch using amber-specific 3-iodo-_L_-tyrosine incorporation. The antidote is synthesized only when 3-iodo-_L_-tyrosine is present in the culture medium. The viability of this strain rapidly decreased with less than a 1 h half-life after removal of 3-iodo-_L_-tyrosine, suggesting that the decay of the antidote causes the host killing by activated colicin E3 in the absence of this unnatural amino acid. The contained strain grew 1.5 times more slowly than the parent strains. The escaper frequency was estimated to be 1.4 mutations (95% highest posterior density 1.1–1.8) per 10^5^ cell divisions. This containment system can be constructed by only plasmid introduction without genome editing, suggesting that this system may be applicable to other microbes carrying toxin-antidote systems similar to that of colicin E3.

## Introduction

In Michael Crichton’s novel “Jurassic Park,” modern technology allowed ancient dinosaurs to come back into existence (Crichton, 1990). Those dinosaurs could not produce the amino acid Lys due to genomic manipulation. The scientists in this novel expected that the dinosaurs could survive only with Lys-feeding and never escape from the park because they would die without the supplement. However, the dinosaurs learned that eating Lys-rich foods, such as chickens or soya beans, allowed them to survive, and then they escaped from the park. One could ask the question: if the auxotrophy was not for Lys but for an amino acid which does not exist in the natural environment, would the dinosaurs have been able to survive after escaping without the supplement?

Although “Jurassic Park” is a science-fiction story, similar problems have been proposed in “the real world” of environmental science. For instance, genetically modified organisms (GMOs) should not be allowed to proliferate in an open environment if their safety has not been certified (Berg et al., 1975). Thus, such GMOs are strictly contained in the laboratory by both physical and biological methods. Harmful invasive species also need to be managed to prevent ecological damage (Marbuah, Gren & McKie, 2014). Pathogens for research use or for live vaccines may pose a serious health risk if the general public is exposed to them, so these organisms must be maintained in controlled areas (Berns, 2014). We are unable to control the proliferation of those organisms once they escape from the laboratory because they autonomously replicate and grow.

Biological containment is a technique used to contain such dangerous organisms in the laboratory (Berg et al., 1975; Curtiss III, 1978). For microbes, this technique involves genetically programing the organisms to grow only in the laboratory and to die in the natural environment. Biological containment has been investigated for genetically-engineered microbes since the initiation of biotechnology research because of their usefulness for environmental detoxification (Paul, Pandey & Jain, 2005; Ramos et al., 2011), biocontrol (Migheli, 2001), and live vaccines (Daniel et al., 2011).

Biological containment systems can be subdivided into active and passive forms (Steidler et al., 2003). Active containment provides control through the conditional expression of toxic genes. A classic example is the bacterium that is engineered to contain the membrane-disruptive toxin gene *hok* controlled by the *trp* promoter (Molin et al., 1987). This bacterium can grow in Trp-containing medium in the laboratory because *hok* expression is repressed. In contrast, the toxin is induced in the absence of Trp, resulting in the death of the bacterium. On the other hand, passive containment is achieved by eliminating essential genes. The contained microbes can grow by complementation of either an auxotrophy or intact gene. For instance, thymidylate synthase (*thyA)*-defective strains cannot grow in an open environment because either thymine or a thymidine supplement is essential for their survival (Ross, O’Gara & Condon, 1990).

In both active and passive containment systems, the life-or-death of the microbes is usually controlled by a substance which occurs in extremely low amounts or is absent in the natural environment. These organisms can survive only by artificially-providing the substance in the laboratory. Outside of the controlled areas, the organisms should die because the substance does not exist or is not present in high enough amounts. Contrary to this expectation, however, some papers have reported that some contained bacteria can survive in the natural environment (Ramos et al., 2011). For example, a passively-contained strain of the bacterium *Rhyzobium meliloti* (*thyA*^−^) became colonized in the presence of alfalfa which could supply either some thymine or thymidine as root exudates (O’Flaherty et al., 1995). A strain of the bacterium *Pseudomonas putida* whose death is induced by proline starvation can proliferate where maize plants secrete proline (van Dillewijn et al., 2004). These reports suggest that the substances which we believe to be rare in the natural environment sometimes exist unexpectedly in specific microenvironments. Thus, the biological containment systems which are controlled by natural substances have a risk of failure similar to the Lys-auxotrophy of dinosaurs in Jurassic Park. In other words, an ideal containment system is where the auxotrophy is for a substance not found anywhere in the natural environment.

Toxin-antidote systems have been reported in many bacteria and in archaea (Yamaguchi, Park & Inouye, 2011). One example is colicin E3 (ColE3) and its cognate antidote (ImmE3) encoded in a plasmid (Jakes & Zinder, 1974; Masaki & Ohta, 1985). Although the free ColE3 kills the host bacterium *Escherichia coli*, ImmE3 neutralizes the toxicity by forming a ColE3-ImmE3 complex. When the plasmid is lost from the bacterium, ColE3 is released from the existing complex because the ImmE3 is more unstable than ColE3, resulting in cell growth inhibition and eventual cell death. Thus, the ColE3-ImmE3 complex is believed to play a role in plasmid maintenance (Thisted et al., 1994).

The sequences of ribosomally-synthesized proteins are encoded in the nucleotide sequences of their genes. A sequence of either three DNA or RNA nucleotides (codon) encodes an amino acid. The codon-amino acid correspondence is highly conserved among organisms. Each codon encodes one of twenty amino acids except for the three specific codons mentioned below (Ambrogelly, Palioura & Söll, 2007). The three specific codons (amber, opal, and ochre) do not code for any amino acids with a few exceptions, and terminate protein translation by binding to peptide chain release factors. Since early in this century, a technique has been developed to incorporate unnatural amino acids other than the twenty standard amino acids in ribosomally-synthesized proteins *in vivo* (Wang et al., 2001; Sakamoto et al., 2002). This technique uses an engineered aminoacyl-tRNA synthetase (aaRS) that specifically recognizes the unnatural amino acid and its cognate amber suppressor tRNA (tRNA_CUA_). The unnatural amino acid is incorporated into proteins at a position encoded by the UAG amber codon in the cells expressing the engineered aaRS/tRNA_CUA_ pair. This unnatural amino acid incorporation system was originally developed for structural and functional analyses, including labeling and functional alteration of proteins (Liu & Schultz, 2010). We focused on the switching function of this system to control translation by the presence/absence of an unnatural amino acid (Minaba & Kato, 2014). The translational switch regulates only the translation of target gene transcripts in which an amber stop codon is inserted next to the AUG translational start codon. In the presence of the unnatural amino acid, translation proceeds beyond the inserted amber stop codon, resulting in the expression of the functional target proteins. In contrast, absence of the unnatural amino acid causes translational termination at the N-terminus, and no functional proteins are obtained. Although leaky expression is often detected in the absence of the unnatural amino acid, such leakage can be reduced by optimization of the expression intensity and balance of the aaRS/tRNA, multiplication of amber stop codons, or by double-regulation with a transcriptional-controlling system. In addition to the all-or-none switching, the translational switch can control the translational efficiency at any intermediate magnitude by adjustment of the unnatural amino acid concentration (Kato, 2015).

Here, we constructed an *Escherichia coli* strain that cannot survive in the absence of an unnatural amino acid, which is a compound not found outside of the laboratory. This study is part of our research project “Construction of engineered organisms auxotrophic for unnatural substances (2013–2015).” This strain has a synthetic essential gene that is expressed only in the presence of the unnatural amino acid that is an artificial essential nutrient. A modified toxin-antidote system is introduced in this bacterium. The antidote is a protein, and an unnatural amino acid translational switch controls the expression of the antidote. The bacterium can survive only when the antidote protein is produced in the presence of the unnatural amino acid in the laboratory. In the natural environment, the bacterium should die due to both the absence of the unnatural amino acid and the accumulation of the toxin. In this paper, we performed a proof-of-concept study for this novel biological containment system using this unnatural amino acid-auxotrophic *E. coli* strain.

## Materials and Methods

### Strains, culture conditions, and transformation

*E. coli* BL21-AI [*F*^−^ *ompT gal dcm lon hsdS*${}_{B}({r}_{B}^{-}{m}_{B}^{-})$ *araB::T7RNAP-tetA*] (Invitrogen) was used throughout this study (Saïda et al., 2006). *E. coli* XL1-blue (carrying an amber suppressor mutation, *supE44*) was also used for plasmid construction (Bullock, Fernandez & Short, 1987). Liquid cultures were grown with rotary shaking at 200 rpm in LB medium (1% bacto tryptone, 0.5% yeast extract, and 1% NaCl). LB agar medium (LB + 2% agar) was used for solid media cultures. All cultures were performed at 37 °C in the presence (3 × 10^−4^ M, approximately equal to 0.1 mg/ml) or absence of the unnatural amino acid, 3-iodo-_L_-tyrosine (IY) (Watanabe Chemical Industries, Ltd., Hiroshima, Japan). Carbenicillin (100 µg/ml) and/or chloramphenicol (50 µg/ml) were added when appropriate. Plasmid-transformation was performed by electroporation using a Gene Pulser (BioRad, Hercules, California, USA).

### Plasmid design

The BL21-AI(IY) strain, which incorporates IY into proteins at a position encoded by the UAG amber codon, was generated by introduction of the plasmid pTYR MjIYRS2-1(D286) MJR1 × 3 (p15A replicon, chloramphenicol resistant) constitutively expressing IYRS and MJR1, originally constructed by K Sakamoto, RIKEN (Fig. S3; Sakamoto et al., 2009). The plasmid pSH350(1amb-immE3) (pMB1 amplicon, ampicillin resistant) encoding *colE3* and an amber-inserted *immE3* was generated from pSH350 which had been originally constructed by H Masaki (Fig. S4; Masaki & Ohta, 1985; Toba, Masaki & Ohta, 1986). An amber stop codon was inserted next to the start codon ATG of *immE3* by inverse PCR using the primers immE3-1amb-s and inv-immE3-as. The primers used in this study are summarized in Table S1. The inverse PCR product was circularized by self-ligation and was transformed into *E. coli* XL1-blue. The transformants were selected on a solid medium containing carbenicillin. Correctly constructed plasmids were selected by colony PCR using the primers amb-immE3-confirm-s and immE3-confirm-as. The sequences of the selected plasmids were confirmed by nucleotide sequencing. The confirmed plasmid was transformed into BL21-AI(IY) to construct BL21AI(IY,1amb-immE3).

### Determination of mutation rates

Mutation rates were estimated by a fluctuation assay (Luria & Delbrück, 1943). A frozen stock of BL21-AI(IY,1amb-immE3) was diluted to prepare an approximately 10 cfu/ml bacterial suspension in a liquid medium containing IY, chloramphenicol, and carbenicillin. Nine parallel cultures (1 ml each) were incubated for 16 h (final OD_590_ = 0.03–0.1). The bacteria were collected by centrifugation (10,000 rpm for 1 min). The bacterial pellets were resuspended in 1 ml of IY-free liquid medium and centrifuged again for washing. This wash was repeated four times to remove IY completely. After washing, the bacterial pellets were resuspended in 1 ml of IY-free liquid medium again, and an aliquot (250 µl) was inoculated onto an IY-free solid medium to detect the “escapers.” Another aliquot was diluted 10^3^ fold, and inoculated onto an IY-containing solid medium to estimate the total number of bacteria. After a 30 h incubation, the number of colonies was counted. The number of colonies did not change with a longer incubation time (Data S4). The mutation rate was calculated by the web tool FALCOR using the Ma-Sandri-Sarkar Maximum Likelihood Estimator (MSS-MLE) method (Hall et al., 2009; Ma, Sandri & Sarkar, 1992). The reliability of this assay was evaluated from both the detected mutation number per culture and the number of parallel cultures (Rosche & Foster, 2000).

### Rate of cell death

BL21-AI(IY,1amb-immE3) was grown to OD_590_ = 0.03–0.1 in 2 ml of IY-containing liquid medium. After washing as described above, the culture was diluted 10^3^-fold in 20 ml of IY-free medium. An aliquot (250 µl) was withdrawn every hour and inoculated onto an IY-containing solid medium. After a 30-h culture, the number of colonies was counted. A single fitted curve was generated using Origin7.

### Growth rate estimation

Growth rates were determined as previously described with some modifications (Minaba & Kato, 2014). Overnight cultures were diluted to an approximate OD_590_ = 0.01. All tested bacterial strains were grown to OD_590_ = 0.05–0.11 in an IY-containing liquid medium. We then measured the change of OD_590_ every 20 min for 2 h. A single fitted curve was generated for each strain using Origin7.

## Results

### Construction of an unnatural amino acid-auxotrophic bacterium

**Figure 1:**
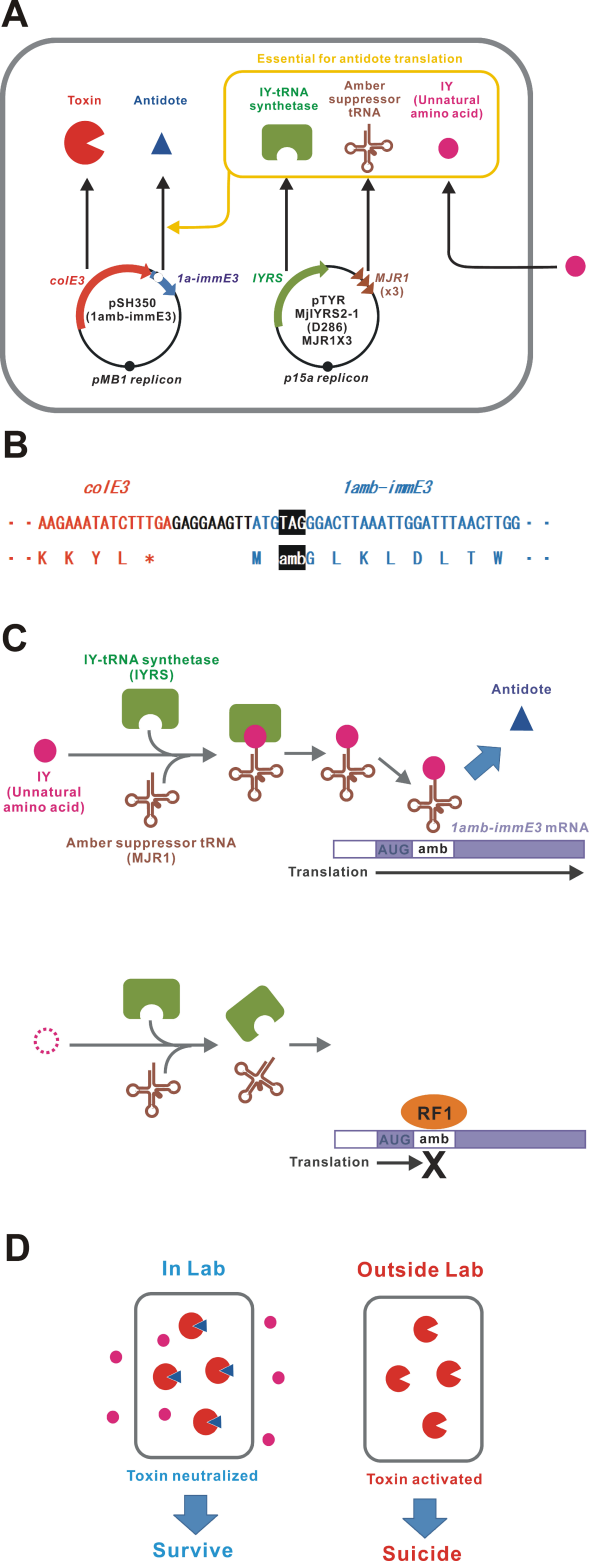
Construction of a bacterium auxotrophic for IY. (A) Schematic of the gene circuit for a synthetic IY-auxotrophy. The IY-auxotrophic *E. coli* strain BL21-AI(IY,1amb-immE3) was constructed by introduction of two plasmids, pSH350(1amb-immE3) and pTYR MjIYRS2-1(D286)MJR1X3. The former plasmid encodes a highly toxic RNase ColE3 and an antidote ImmE3. *immE3* was modified as described below. IYRS and MJR1 genes are encoded in the latter plasmid. The translation of ImmE3 is regulated by the IY-controlling translational switch. (B) Alteration of *immE3*. An amber stop codon was inserted next to the translation start codon ATG. This inserted amber codon is the site of IY incorporation. (C) IY-dependent translation of ImmE3. IY is incorporated at the site of the inserted amber stop codon in the presence of IYRS and MJR1, resulting in the successful translation of ImmE3 encoded downstream of the amber codon. The translation is interrupted in the absence of IY. RF1, peptide chain release factor 1. (D) Expected responses. The antidote (ImmE3) is produced in the presence of IY (in the laboratory), thus neutralizing the toxin (ColE3), and the host bacterium survives. In contrast, the antidote is not produced in the absence of IY (in the natural environment), thus the toxin is expressed, and the host bacterium is killed.

An unnatural amino acid-auxotrophic *E. coli* strain was constructed by introduction of two plasmids (Fig. 1A). One plasmid encoded the toxin-antidote pair ColE3-ImmE3. ColE3 is a highly toxic RNase that can kill the host bacterium with only a few molecules (Lazzaroni, Dubuisson & Vianney, 2002; Bowers et al., 2004). ImmE3 forms a complex with ColE3 and inhibits the RNase activity (Yajima et al., 1992). In this plasmid, an amber stop codon was inserted next to the *immE3* translation start codon ATG (Fig. 1B). Toxin-antidote systems, such as ColE3-ImmE3, are good examples of selfish genetic elements (Inglis et al., 2013). We speculate that the relative expression levels of those genes may be optimally adjusted to maintain themselves for many generations with minimum consequences to the host. Therefore, the gene organization and sequences of *colE3-immE3* and their gene-regulatory regions were unchanged, except for the amber insertion in *immE3*. Another plasmid expresses the aaRS/tRNA pair (IYRS/MJR1) for amber-specific incorporation of the unnatural amino acid 3-iodo-_L_-tyrosine (IY) (Sakamoto et al., 2009). This IY-incorporation system acts as a translational switch for amber-inserted target genes (Minaba & Kato, 2014; Kato, 2015). BL21-AI was selected as a host strain because the translational switch using IY incorporation was well characterized in this strain (Minaba & Kato, 2014; Kato, 2015). We constructed the BL21-AI strain carrying these two plasmids and designated it BL21-AI(IY,1amb-immE3). IY from the culture medium is taken up into the intracellular space of the bacteria. Intracellular IY is ribosomally incorporated in ImmE3 at the position of the inserted amber codon, resulting in the expression of functional ImmE3 that represses the ColE3 toxicity (Figs. 1C and 1D). Therefore, BL21-AI(IY,1amb-immE3) can survive in an IY-containing medium. In contrast, the absence of IY interrupts the production of ImmE3, resulting in the death of BL21-AI(IY,1amb-immE3) due to ColE3 toxicity.

**Figure 2:**
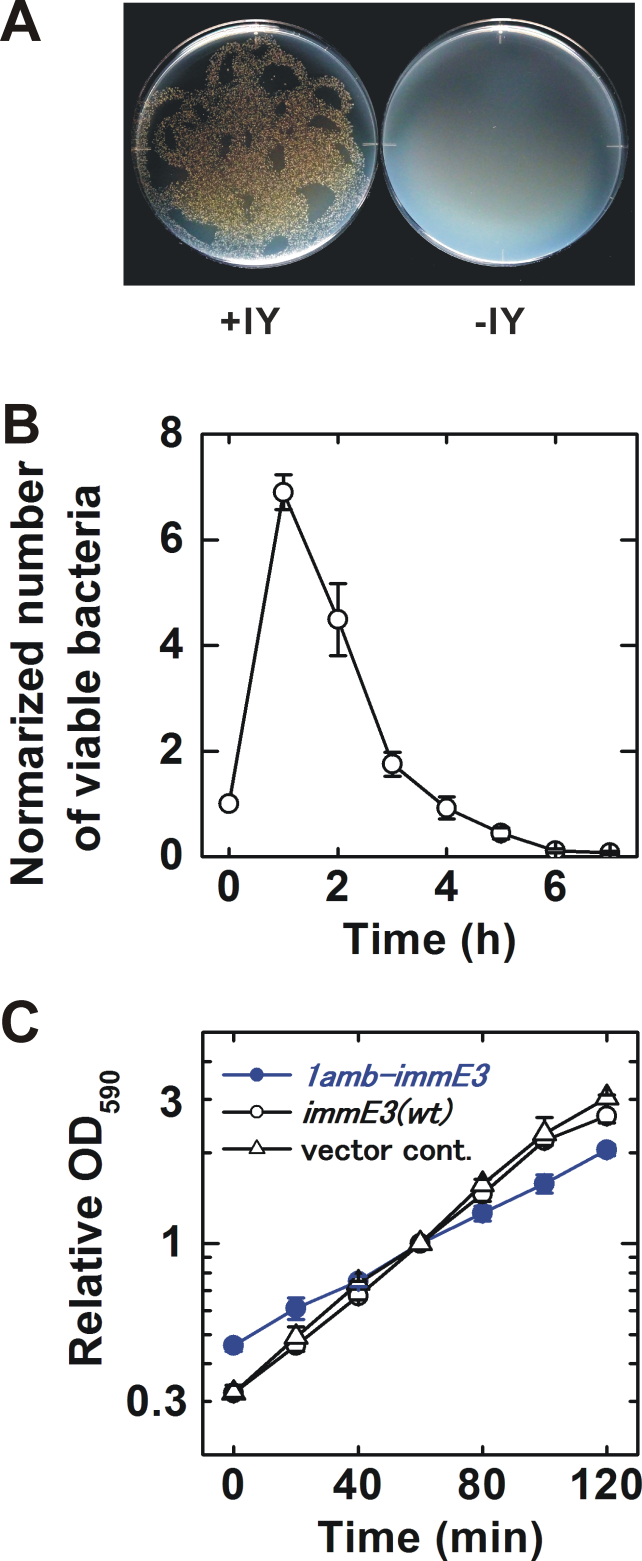
Characterization of the constructed bacterium. (A) Evaluation of IY-auxotrophy. Ten thousand viable BL21-AI(IY,1amb-immE3) bacterial cells were inoculated as several drops onto a solid medium. Then the drops were spread by inclining the plates. The resulting “twiggy pattern” of colonies was an artifact of this incomplete coverage of the plate. Left, an IY-containing medium; right, an IY-free medium. (B) Rate of killing. IY in the medium was removed at time 0. The number of viable cells is normalized (the value at time 0 = 1). *n* = 4 independent experiments using separate bacterial cultures. (C) Growth curves. BL21-AI(IY,1amb-immE3), the parent strain carrying pSH350, and a vector control carrying pBR322 instead of pSH350 were tested. All tested strains maintain pTYR MjIYRS2-1(D286)MJR1X3. The OD_590_ was normalized (the value at 60 min = 1). *n* = 3 independent experiments using separate bacterial cultures. Data (B and C) are shown as means ± SEM.

The IY-auxotrophy of BL21-AI(IY,1amb-immE3) was experimentally evaluated (Fig. 2A). The bacterium was inoculated onto either an IY-free or IY-containing solid medium, after stringent washes to remove IY completely. Many viable colonies (8.5 ×10^3^ cfu) were observed on the IY-containing medium. In contrast, no colonies were detected on the IY-free medium, indicating that IY is essential for the survival of BL21-AI(IY,1amb-immE3). However, approximately 1.5 × 10^2^ escapers were detected when we inoculated 2.3 × 10^6^ of bacteria (Table 1 and Data S3).

**Table 1:** Calculation of escape frequency. Escaper generation rates were estimated by a fluctuation assay^a^ using the web tool FALCOR^b^.

	MSS Maximum likelihood method^c^
**Method**	
Mutations (m)	32.5
Mutation rate (per 10^5^)	1.4
95% CI range (Upper Bound)	1.8
95% CI range (Lower Bound)	1.1
95% CI difference (Upper Difference)	0.4
95% CI difference (Lower Difference)	0.4
**Reliability test** ^d^	
Avaraged number of inoculated bacteria (N)	2.3 × 10^6^
Avaraged number of escapers (m)	148.9
Number of cultures required to achieve a theoretical precision of 20% for MSS-ML	<2
Number of cultures in this experiment	9
Result	Reliable

**Notes.**

a Luria & Delbrück (1943).

b Hall et al. (2009).

c Ma, Sandri & Sarkar (1992).

d Rosche & Foster (2000).

### Rate of killing after IY removal

Contained bacteria may make an impact on the natural environment if they can survive for a significantly long time after removal of their auxotropic substances. We determined the rate of killing after IY-removal for BL21-AI(IY,1amb-immE3) (Fig. 2B and Data S1). The number of viable bacteria increased until 1 h after IY-removal. At 2 h after the removal of IY, the viability rapidly decreased. The half-life was estimated to be 49.2 ± 7.2 min (Fig. S1).

### Growth rate

The growth rate is an important characteristic related to competitiveness for survival. The growth rates of BL21-AI(IY,1amb-immE3) and related strains were determined and compared with each other (Fig. 2C and Data S2). BL21-AI(IY,1amb-immE3) grew at a slower rate (doubling time = 59.8 ±9.6 min) than that of the parent strain carrying no amber-inserted *immE3* (40.5 ± 2.6 min) and of a vector control strain (37.9 ± 1.6 min) (Fig. S2).

### Escape frequency

An important problem in biological containment is emergence of escapers by genetic mutations. Only a few escapers can lead to an uncontrolled proliferation of contained microbes. Therefore, we estimated the frequency of escaper emergence for BL21-AI(IY,1amb-immE3) using a fluctuation assay (Table 1 and Data S3). The frequency was estimated to be 1.4 mutations (95% highest posterior density 1.1–1.8) per 10^5^ cell divisions. This means that 71 thousand cell divisions will generate one escaper.

## Discussion

In this study, we constructed the *E. coli* strain BL21-AI(IY,1amb-immE3) which cannot survive in the absence of the unnatural amino acid IY. The biological containment system used for this strain involves a ColE3-dependent killing mechanism, indicating that it is an active containment system. Although *immE3* is introduced from outside of the bacterium, this strain cannot survive without this gene, suggesting that immE3 is a synthetic essential gene. In this strain, ImmE3 is produced only in the presence of IY. IY is not only a regulatory molecule, such as isopropyl *β*-_D_-1-thiogalactopyranoside (IPTG) for the *lac* promoter control, but also a building block for the formation of an aminoacyl-tRNA and target proteins, indicating that IY is a synthetic essential “nutrient” (Minaba & Kato, 2014). This containment system therefore involves complementation of an auxotrophy, suggesting that it is also a passive containment system.

In the usual passive containment systems, an auxotrophy is conferred by disruption of genes in the synthetic pathway for an essential metabolite (Steidler et al., 2003). Such auxotrophy is limited to natural metabolites, and is not possible for substances that do not occur in the natural environment. The unnatural amino acid IY does not exist in the natural environment, suggesting that the auxotrophy for the unnatural amino acid cannot be constructed using classic methods. The IY-auxotrophy was constructed by the introduction of an IY-specific aaRS/tRNA_CUA_ pair and an insertion of an amber codon into a target gene. This engineered auxotrophy is a promising method to generate organisms that require for their survival substances that do not naturally occur, and thus is a improved biological containment system which is free of the Jurassic Park-type risk. However, most unnatural amino acids are expensive, indicating that cost may be a limitation for the application of this method (Minaba & Kato, 2014).

BL21-AI(IY,1amb-immE3) was constructed only by the introduction of two plasmids and without genome editing. Although plasmids are useful for easy gene introduction, their use has a problem for environmental applications because of possible horizontal gene transfer to other bacteria (Ramos et al., 2011). In the case of BL21-AI(IY,1amb-immE3), the plasmid carrying *colE3-immE3* is not transmittable because the *immE3* in this plasmid is a loss-of-function mutant if the recipient strains are not amber-suppressor mutants. Even if an amber-suppressor strain contains the plasmid, the *colE3-immE3* is a native *E. coli* plasmid gene cluster in which the natural sequences are kept except for an insertion of the amber codon. In addition, the substrates of IY-specific aaRS/tRNA_CUA_, which is encoded in another plasmid, do not exist in the natural environment with a few exceptions such as the thyroid cells and the skeleton of sponges (Kato, 2015), suggesting that the genetic and ecological impact may be minimal if those genetic parts are released into the natural environment. Although the selection markers (chloramphenicol- and ampicillin-resistant genes) are definitively problematic, we can avoid those genes by alternative methods for selection of strains carrying those plasmids, such as plasmid-complementation for auxotrophy which is caused by genomic gene disruptions (Wright et al., 2014).

The target gene products that are regulated by the IY-controlling translational switch require the incorporation of IY. IY-incorporation may cause functional alterations in some target proteins. The IY-incorporated ImmE3 functions to neutralize the ColE3 toxicity. The chimeric ImmE3 whose N-terminal region is replaced by that of ImmE6 maintains its protection against ColE3, suggesting that the N-terminal region is not essential (Masaki et al., 1991). This report agrees with our observation. However, the growth rate of BL21-AI(IY,1amb-immE3) was slower than that of its parent strain. Although IY-incorporation also suppresses natural amber stop codons that are contained in host genomes, the growth rate of BL21-AI was not affected (Minaba & Kato, 2014; Kato, 2015), suggesting that this slower growth rate of BL21-AI(IY,1amb-immE3) might be caused by reduction of the immunity and/or expression level of amber-inserted *immE3.* RF1 competition might be a possible mechanism (Yarus et al., 1986).

BL21-AI(IY,1amb-immE3) continued to proliferate even 1 h after IY removal. This could be due to intracellular IY accumulation. The termination of synthesis and degradation of ImmE3 are assumed to be the cause of the rapid decline in viability within 2 h after the removal of IY. Leakage translation which is several percent of the maximum translation was observed in the IY-controlling translational switch, probably due to natural amino acid mischarges in MJR1 (Minaba & Kato, 2014; Kato, 2015). Such incomplete repression of ImmE3 is still sufficient to induce the killing of the host by ColE3 in this system.

The frequency of emergence of escapers was 1.4 × 10^−5^ mutations/cell/generation. This value is one order higher than that reported for an early plasmid containment system using the conditional expression of *relF* controlled by the inducible *lac* promoter (Knudsen & Karlström, 1991). If the biological containment system reported here is used alone, it is not a practical system in the present form (Schmidt & de Lorenzo, 2012). Furthermore, the efficacy of biological containment systems using a single containment gene is highly insufficient. Higher containment efficacy is usually achieved by a multi-layer combination of containment systems (Ronchel & Ramos, 2001; Torres et al., 2003; Rovner et al., 2015). Our system should also be improved by such a combination use to repress the emergence of escapers completely. In other words, this active containment system is promising as a part of an integrated containment system comprised of other containment mechanisms as reported recently (Gallagher et al., 2015; Cai et al., 2015). In addition, the mechanisms of escaper emergence should also be analyzed for system improvement. Mutational inactivation of *colE3*, natural amber suppressors, and non-synonymous mutations in the inserted amber codon of *immE3* may be involved.

Early this year, passive containment systems using conditional expression of genomic essential genes depending on unnatural amino acids were reported (Mandell et al., 2015; Rovner et al., 2015). These systems have achieved an excellent containment efficacy by controlling multiple containment genes. They used the genomically-recoded *E. coli* strain C321.AA (GRO) as a host (Lajoie et al., 2013). GRO has a largely edited genome whose amber codons (TAG) were all substituted by ochre codons (TAA). Moreover, the peptide chain release factor gene *prfA* was deleted for efficient incorporation of unnatural amino acids. These systems could therefore not be used for other microbes in which the techniques for genome editing have not been adequately developed. In contrast, the containment system shown here was constructed by introduction of only two plasmids with a minimum alteration of the natural sequence, i.e., only an amber insertion into *immE3*. Toxin-antidote systems, including type II, type IV, and type V toxin-antidote systems with protein antidotes (Shuster & Bertram, 2013), such as ColE3-ImmE3 used in this study, have been reported in almost all bacteria and in many archaea (Yamaguchi, Park & Inouye, 2011). Plasmids have also been identified in both bacteria and archaea (Thomas & Summers, 2008; Wang et al., 2015), suggesting that this system could be easily used for other bacteria and for archaea.

## Supplemental Information

10.7717/peerj.1247/supp-1 Figure S1 Estimation of the half-life The half-life of the viability for BL21-AI(IY,1amb-immE3) was estimated in the absence of IY. A single fitted curve was generated using an exponential decrease model. The half-life was calculated from the equation for the fitted curve.

10.7717/peerj.1247/supp-2 Figure S2 Estimation of the growth rates The growth rates were estimated for BL21-AI(IY,1amb-immE3), the parent strain carrying pSH350, and for a vector control carrying pBR322 instead of pSH350. A single fitted curve was generated for each strain using an exponential increase model. The growth rates were calculated from the equations for the fitted curves.

10.7717/peerj.1247/supp-3 Figure S3 Sequence of the plasmid pTYR MjIYRS2-1(D286) MJR1x3 This plasmid was originally constructed by Kensaku Sakamoto, RIKEN (Sakamoto et al., 2009).

10.7717/peerj.1247/supp-4 Figure S4 Sequence of the plasmid pSH350(1amb-immE3) This plasmid was generated from pSH350 which had been originally constructed by Haruhiko Masaki and others (Masaki & Ohta, 1985; Toba, Masaki & Ohta, 1986).

10.7717/peerj.1247/supp-5 Table S1 List of PCR primers

10.7717/peerj.1247/supp-6 Data S1 Data set for Fig. 2B

10.7717/peerj.1247/supp-7 Data S2 Data set for Fig. 2C

10.7717/peerj.1247/supp-8 Data S3 Data set for fluctuation assay

10.7717/peerj.1247/supp-9 Data S4 Effect of longer incubation
