# Supplementary material for: An engineered bacterium auxotrophic for an unnatural amino acid: a novel biological containment system
Source: PeerJ. 2015 Sep 15;3:e1247. doi: 10.7717/peerj.1247 (PMC4579030; doi:10.7717/peerj.1247)
Supplement: Figure S3 — This plasmid was originally constructed by Kensaku Sakamoto, RIKEN (Sakamoto et al., 2009). [file peerj-03-1247-s003.pdf]

pSH350(1amb-immE3)

0001 GTCAACTCGG TTTTAATCAG ACCTGGCATG AGTGGAAGCG GGACGAACAG

*Hinc*II

→ ColE3-CA38 derived sequence

← pBR328 plasmid backbone

0051 CACAGGCAAC AACAACGCCG CCCCGGGCAC TTCCGGGGCA TGAGTATGTG
0101 ATATCCGGGG CTGCACCCCG GACCCCGCCA ACACATCACG GGCCACAAAA
0151 TTTTTTGTGG CCCGCTCTGC GTTTTCTAAG TGTTATCCCT CCTGATTTCT
0201 AAAAAATTTT CCACCTGAAC TTGACAGAAA AAACGATGAC GAGTACTTTT
0251 TGATCTGTAC ATAAACCCAG TGGTTTTATG TACAGTATTA ATCGTGTAAT
0301 CAATTGTTTT AACGCTTAAA AGAGGGAATT TTTATGAGCG GTGGCGATGG

colE3

0351 ACGCGGCCAT AACACGGGCG CGCATAGCAC AAGTGGTAAC ATTAATGGTG
0401 GCCCGACCGG GCTTGGTGTA GGTGGTGGTG CTTCTGATGG CTCCGGATGG
0451 AGTTCGGAAA ATAACCCGTG GGGTGGTGGT TCCGGTAGCG GCATTCACTG
0501 GGGTGGTGGT TCCGGTCATG GTAATGGCGG GGGGAATGGT AATTCCGGTG
0551 GTGGTTCGGG AACAGGCGGT AATCTGTCAG CAGTAGCTGC GCCAGTGGCA
0601 TTTGGTTTTC CGGCACTTTC CACTCCAGGA GCTGGCGGTC TGGCGGTCAG
0651 TATTTCAGCG GGAGCATTAT CGGCAGCTAT TGCTGATATT ATGGCTGCCC
0701 TGAAAGGACC GTTTAAATTT GGTCTTTGGG GGGTGGCTTT ATATGGTGTA
0751 TTGCCATCAC AAATAGCGAA AGATGACCCC AATATGATGT CAAAGATTGT
0801 GACGTCATTA CCCGCAGATG ATATTACTGA ATCACCTGTC AGTTCATTAC
0851 CTCTCGATAA GGCAACAGTA AACGTAAATG TTCGTGTTGT TGATGATGTA
0901 AAAGACGAGC GACAGAATAT TTCGGTTGTT TCAGGTGTTC CGATGAGTGT
0951 TCCGGTGGTT GATGCAAAAC CTACCGAACG TCCGGGTGTT TTTACGGCAT
1001 CAATTCCAGG TGCACCTGTT CTGAATATTT CAGTTAATAA CAGTACGCCA
1051 GCAGTACAGA CATTAAGCCC AGGTGTTACA AATAATACTG ATAAGGATGT
1101 TCGCCCGGCA GGATTTACTC AGGGTGGTAA TACCAGGGAT GCAGTTATTC
1151 GATTCCCGAA GGACAGCGGT CATAATGCCG TATATGTTTC AGTGAGTGAT
1201 GTTCTTAGCC CTGACCAGGT AAAACAACGT CAAGATGAAG AAAATCGCCG
1251 TCAGCAGGAA TGGGATGCTA CGCATCCGGT TGAAGCGGCT GAGCGAAATT
1301 ATGAACGCGC GCGTGCAGAG CTGAATCAGG CAAATGAAGA TGTTGCCAGA

1351 AATCAGGAGC GACAGGCTAA AGCTGTTCAG GTTTATAATT CGCGTAAAAG
1401 CGAACTTGAT GCAGCGAATA AAACTCTTGC TGATGCAATA GCTGAAATAA
1451 AACAATTTAA TCGATTTGCC CATGACCCAA TGGCTGGCGG TCACAGAATG
1501 TGGCAAATGG CCGGGCTTAA AGCCCAGCGG GCGCAGACGG ATGTAAATAA
1551 TAAGCAGGCT GCATTTGATG CTGCTGCAAA AGAGAAGTCA GATGCTGATG
1601 CTGCATTGAG TTCTGCTATG GAAAGCAGGA AGAAGAAAGA AGATAAGAAA
1651 AGGAGTGCTG AAAATAATTT AAACGATGAA AAGAATAAGC CCAGAAAAGG
1701 TTTTAAAGAT TACGGGCATG ATTATCATCC AGCTCCGAAA ACTGAGAATA
1751 TTAAAGGGCT TGGTGATCTT AAGCCTGGGA TACCAAAAAC ACCAAAGCAG
1801 AATGGTGGTG GAAAACGCAA GCGCTGGACT GGAGATAAAG GGCGTAAGAT
1851 TTATGAGTGG GATTCTCAGC ATGGTGAGCT TGAGGGGTAT CGTGCCAGTG
1901 ATGGTCAGCA TCTTGGCTCA TTTGACCCTA AAACAGGCAA TCAGTTGAAA
1951 GGTCCAGATC CGAAACGAAA TATCAAGAAA TATCTTTGAG AGGAAGTTAT

immE3

2001 GTAGGGACTT AAATTGGATT TAACTTGGTT TGATAAAAGT ACAGAAGATT

amber

2051 TTAAGGGTGA GGAGTATTCA AAAGATTTTG GAGATGACGG TTCAGTTATG
2101 GAAAGTCTAG GTGTGCCTTT TAAGGATAAT GTTAATAACG GTTGCTTTGA
2151 TGTTATAGCT GAATGGGTAC CTTTGCTACA ACCATACTTT AATCATCAAA
2201 TTGATATTTC CGATAATGAG TATTTTGTTT CGTTTGATTA TCGTGATGGT
2251 GATTGGTGAT CAAATATTAT CAGGGATGAG TTGATATACG GGCTTCTAGT
2301 GTTCATGGAT GAACGCTGGA GCCTCCAAAT GTAGAAATGT TATATTTTTT
2351 ATTGAGTTCT TGGTTATAAT TGCTCCGCAA TGATTTAAAT AAGCATTATT
2401 TAAAACATTC TCAGGAGAGG TGAAGGTGGA GCTAAAAAAA AGTATTGGTG

immE8

2451 ATTACACTGA AACCGAATTC AAAAAATTTA TTGAAGACAT CATCAATTGT
2501 GAAGGTGATG AAAAAAAACA GGATGATAAC CTCGAGTATT TTATAAATGT
2551 TACTGAGCAT CCTAGTGGTT CTGATCTGAT TTATTACCCA GAAGGTAATA
2601 ATGATGGTAG CCCTGAAGGT GTTATTAAAG AGATTAAAGA ATGGCGAGCC
2651 GCTAACGGTA AGTCAGGATT TAAACAGGGC TGAAATATGA ATGCCGGTTG
2701 TTTATGGATG AATGGCTGGC ATTCTTTCAC AACAAGGAGT CGTTATGAAA

Lysis protein

2751 AAAATAACAG GGATTATTTT ATTGCTTCTT GCAGTCATTA TTCTGTCTGC
2801 ATGTCAGGCA AACTATATCC GGGATGTTCA GGGCGGGACC GTATCTCCGT
2851 CATCAACAGC TGAAGTGACC GGATTAGCAA CGCAGTAACC CGAAATCCTC
2901 TTTGACAAAA ACAAAGCGTG TCAGGCTGAT TCTGATGCGC TTTTTTTTTG
2951 AAATGTCACA AAAATTCCAT GTGGGAGATG GGATCTAAAA TCCTCGTGCA
3001 GAACTTTCCA TCCAGGGGGA GAAAACTTGT CGTTTTGAGC CGTTCGGTGT
3051 TCAGAACGCA CGAAACCGAA TTCAGGTGGC ACTTTTCGGG GAAATGTGCG

***Eco*RI**

ColE3-CA38 derived sequence ←

→ pBR328 plasmid backbone

3101 CGGAACCCCT ATTTGTTTAT TTTTCTAAAT ACATTCAAAT ATGTATCCGC
3151 TCATGAGACA ATAACCCTGA TAAATGCTTC AATAATATTG AAAAAGGAAG
3201 AGTATGAGTA TTCAACATTT CCGTGTCGCC CTTATTCCCT TTTTTGCGGC
3251 ATTTTGCCTT CCTGTTTTTG CTCACCCAGA AACGCTGGTG AAAGTAAAAG
3301 ATGCTGAAGA TCAGTTGGGT GCACGAGTGG GTTACATCGA ACTGGATCTC
3351 AACAGCGGTA AGATCCTTGA GAGTTTTCGC CCCGAAGAAC GTTTTCCAAT
3401 GATGAGCACT TTTAAAGTTC TGCTATGTGG CGCGGTATTA TCCCGTGTTG
3451 ACGCCGGGCA AGAGCAACTC GGTCGCCGCA TACACTATTC TCAGAATGAC
3501 TTGGTTGAGT ACTCACCAGT CACAGAAAAG CATCTTACGG ATGGCATGAC
3551 AGTAAGAGAA TTATGCAGTG CTGCCATAAC CATGAGTGAT AACACTGCGG
3601 CCAACTTACT TCTGACAACG ATCGGAGGAC CGAAGGAGCT AACCGCTTTT
3651 TTGCACAACA TGGGGGATCA TGTAACTCGC CTTGATCGTT GGGAACCGGA
3701 GCTGAATGAA GCCATACCAA ACGACGAGCG TGACACCACG ATGCCTGCAG
3751 CAATGGCAAC AACGTTGCGC AAACTATTAA CTGGCGAACT ACTTACTCTA
3801 GCTTCCCGGC AACAATTAAT AGACTGGATG GAGGCGGATA AAGTTGCAGG
3851 ACCACTTCTG CGCTCGGCCC TTCCGGCTGG CTGGTTTATT GCTGATAAAT
3901 CTGGAGCCGG TGAGCGTGGG TCTCGCGGTA TCATTGCAGC ACTGGGGCCA
3951 GATGGTAAGC CCTCCCGTAT CGTAGTTATC TACACGACGG GGAGTCAGGC
4001 AACTATGGAT GAACGAAATA GACAGATCGC TGAGATAGGT GCCTCACTGA
4051 TTAAGCATTG GTAACTGTCA GACCAAGTTT ACTCATATAT ACTTTAGATT
4101 GATTTAAAAC TTCATTTTTA ATTTAAAAGG ATCTAGGTGA AGATCCTTTT
4151 TGATAATCTC ATGACCAAAA TCCCTTAACG TGAGTTTTCG TTCCACTGAG
4201 CGTCAGACCC CGTAGAAAAG ATCAAAGGAT CTTCTTGAGA TCCTTTTTTT

pMB1 origin

4251 CTGCGCGTAA TCTGCTGCTT GCAAACAAAA AAACCACCGC TACCAGCGGT
4301 GGTTTGTTTG CCGGATCAAG AGCTACCAAC TCTTTTTCCG AAGGTAACTG
4351 GCTTCAGCAG AGCGCAGATA CCAAATACTG TCCTTCTAGT GTAGCCGTAG
4401 TTAGGCCACC ACTTCAAGAA CTCTGTAGCA CCGCCTACAT ACCTCGCTCT
4451 GCTAATCCTG TTACCAGTGG CTGCTGCCAG TGGCGATAAG TCGTGTCTTA
4501 CCGGGTTGGA CTCAAGACGA TAGTTACCGG ATAAGGCGCA GCGGTCGGGC
4551 TGAACGGGGG GTTCGTGCAC ACAGCCCAGC TTGGAGCGAA CGACCTACAC
4601 CGAACTGAGA TACCTACAGC GTGAGCTATG AGAAAGCGCC ACGCTTCCCG
4651 AAGGGAGAAA GGCGGACAGG TATCCGGTAA GCGGCAGGGT CGGAACAGGA
4701 GAGCGCACGA GGGAGCTTCC AGGGGGAAAC GCCTGGTATC TTTATAGTCC
4751 TGTCGGGTTT CGCCACCTCT GACTTGAGCG TCGATTTTTG TGATGCTCGT
4801 CAGGGGGGCG GAGCCTATGG AAAAACGCCA GCAACGCGGC CCGAGATGCG
4851 CCGCGTGCGG CTGCTGGAGA TGGCGGACGC GATGGATATG TTCTGCCAAG
4901 GGTTGGTTTG CGCATTCACA GTTCTCCGCA AGAATTGATT GGCTCCAATT
4951 CTTGGAGTGG TGAATCCGTT AGCGAGGTGC CGCCGGCTTC CATTCAGGTC
5001 GAGGTGGCCC GGCTCCATGC ACCGCGACGC AACGCGGGGA GGCAGACAAG
5051 GTATAGGGCG GCGCCTACAA TCCATGCCAA CCCGTTCCAT GTGCTCGCCG
5101 AGGCGGCATA AATCGCCGTG ACGATCAGCG GTCCAGTGAT CGAAGTTAGG
5151 CTGGTAAGAG CCGCGAGCGA TCCTTGAAGC TGTCCCTGAT GGTCGTCATC
5201 TACCTGCCTG GACAGCATGG CCTGCAACGC GGGCATCCCG ATGCCGCCGG
5251 AAGCGAGAAG AATCATAATG GGGAAGGCCA TCCAGCCTCG CGTCGCGAAC
5301 GCCAGCAAGA CGTAGCCCAG CGCGTCGGCC GCCATGCCGG CGATAATGGC
5351 CTGCTTCTCG CCGAAACGTT TGGTGGCGGG ACCAGTGACG AAGGCTTGAG
5401 CGAGGGCGTG CAAGATTCCG AATACCGCAA GCGACAGGCC GATCATCGTC
5451 GCGCTCCAGC GAAAGCGGTC CTCGCCGAAA ATGACCCAGA GCGCTGCCGG
5501 CACCTGTCCT ACGAGTTGCA TGATAAAGAA GACAGTCATA AGTGCGGCGA
5551 CGATAGTCAT GCCCCGCGCC CACCGGAAGG AGCTGACTGG GTTGAAGGCT
5601 CTCAAGGGCA TCG

//
