# Supplementary material for: An engineered bacterium auxotrophic for an unnatural amino acid: a novel biological containment system
Source: PeerJ. 2015 Sep 15;3:e1247. doi: 10.7717/peerj.1247 (PMC4579030; doi:10.7717/peerj.1247)
Supplement: Figure S4 — This plasmid was generated from pSH350 which had been originally constructed by Haruhiko Masaki and others (Masaki & Ohta, 1985; Toba, Masaki & Ohta, 1986). [file peerj-03-1247-s004.pdf]

pTYR MjIYRS2-1(D286)MJR1×3

0001 TGATGCGTGG AAGATTGATC GTCTTGCACC CTGAAAAGAT GCAAAAATCT

E. coli tyrS promoter(1)

0051 TGCTTTAATC GCTGGTACTC CTGATTCTGG CACTTTATTC TATGTCTCTT
0101 TCGCATCTGG CGAAAAGTCG TGTACCGGCA AAGGTGCAGT CGTTATATAC
0151 TTGGAGATTC ATATGTTCCA GCTTGATGCG TGGAAGATTG ATCGTCTTGC
0201 ACCCTGAAAA GATGCAAAAA TCTTGCTTTA ATCGCTGGTA CTCCTGATTC

E. coli tyrS promoter(2)

0251 TGGCACTTTT ATTCTATGTC TCTTTCGCAT CTGGCGAAAA GTCGTGTACC
0301 GGCAAGGTGC AGTCGTTATA TACTTGAGAT TCATATGGAC GAATTTGAAA

Iodotyrosyl-tRNA synthetase (IYRS)

0351 TGATAAAGAG AAACACATCT GAAATTATCA GCGAGGAAGA GTTAAGAGAG
0401 GTTTTAAAAA AAGATGAAAA ATCTGCTTAC ATAGGTTTTG AACCAAGTGG
0451 TAAAATACAT TTAGGGCATT ATCTCCAAAT AAAAAAGATG ATTGATTTAC
0501 AAAATGCTGG ATTTGATATA ATTATATTGT TGGCTGATTT AGCCGCCTAT
0551 TTAAACCAGA AAGGAGAGTT GGATGAGATT AGAAAAATAG GAGATTATAA
0601 CAAAAAAGTT TTTGAAGCAA TGGGGTTAAA GGCAAAATAT GTTTATGGAA
0651 GTGAATTCCA GCTTGATAAG GATTATACAC TGAATGTCTA TAGATTGGCT
0701 TTAAAAACTA CCTTAAAAAG AGCAAGAAGG AGTATGGAAC TTATAGCAAG
0751 AGAGGATGAA AATCCAAAGG TTGCTGAAGT TATCTATCCA ATAATGCAGG
0801 TTAATACCAG TCATTATTTA GGCGTTGATG TTGCAGTTGG AGGGATGGAG
0851 CAGAGAAAAA TACACATGTT AGCAAGGGAG CTTTTACCAA AAAAGGTTGT
0901 TTGTATTCAC AACCCTGTCT TAACGGGTTT GGATGGAGAA GGAAAGATGA
0951 GTTCTTCAAA AGGGAATTTT ATAGCTGTTG ATGACTCTCC AGAAGAGATT
1001 AGGGCTAAGA TAAAGAAAGC ATACTGCCCA GCTGGAGTTG TTGAAGGAAA
1051 TCCAATAATG GAGATAGCTA AATACTTCCT TGAATATCCT TTAACCATAA
1101 AAAGGCCAGA AAAATTTGGT GGAGATTTGA CAGTTAATAG CTATGAGGAG
1151 TTAGAGAGTT TATTTAAAAA TAAGGAATTG CATCCAATGC GTTTAAAAAA
1201 TGCTGTAGCT GAAGAACTTA TAAAGATTTT AGAGCCAATT AGAAAGAGAT
1251 TATAAAGATC TGGATCCTCT ACGCCGGACG CATCGTGGCC GGCATCACCG
1301 GCGCCACAGG TGCGGTTGCT GGCGCCTATA TCGCCGACAT CACCGATGGG

```
1351  GAAGATCGGG CTCGCCACTT CGGGCTCATG AGGCATGCGG CGCCGCTTCT
1401  TTGAGCGAAC GATCAAAAAT AAGTGGCGCC CCATCAAAAA AATATTCTCA
                                        lpp promoter

1451  ACATAAAAAA CTTTGTGTAA TACTTGTAAC GCTGCCATCA GACGCATTCC
                                      MJR1 amber suppressor tRNA(1)

1501  GGCGGTAGTT CAGCAGGGCA GAACGGCGGA CTCTAAATCC GCAGGTCGCT
1551  GGTTCAAATC CGGCCCGCCG GACCATTTAT CACAGACGCA TTCCGGCGGT
                                      MJR1 amber suppressor tRNA(2)

1601  AGTTCAGCAG GGCAGAACGG CGGACTCTAA ATCCGCAGGT CGCTGGTTCA
1651  AATCCGGCCC GCCGGACCAT TTATCACAGA CGCATTCCGG CGGTAGTTCA
                                      MJR1 amber suppressor tRNA(3)

1701  GCAGGGCAGA ACGGCGGACT CTAAATCCGC AGGTCGCTGG TTCAAATCCG
1751  GCCCGCCGGA CCATTTATCA CAGATTGGAA ATTTTTGATC CTTAGCGAAA
1801  GCTAAGGATT TTTTTTAGTC GACCGATGCC CTTGAGAGCC TTCAACCCAG
1851  TCAGCTCCTT CCGGTGGGCG CGGGGCATGA CTATCGTCGC CGCACTTATG
1901  ACTGTCTTCT TTATCATGCA ACTCGTAGGA CAGGTGCCGG CAGCGCTCTG
1951  GGTCATTTTC GGCGAGGACC GCTTTCGCTG GAGCGCGACG ATGATCGGCC
2001  TGTCGCTTGC GGTATTCGGA ATCTTGCACG CCCTCGCTCA AGCCTTCGTC
2051  ACTGGTCCCG CCACCAAACG TTTCGGCGAG AAGCAGGCCA TTATCGCCGG
2101  CATGGCGGCC GACGCGCTGG GCTACGTCTT GCTGGCGTTC GCGACGCGAG
2151  GCTGGATGGC CTTCCCCATT ATGATTCTTC TCGCTTCCGG CGGCATCGGG
2201  ATGCCCGCGT TGCAGGCCAT GCTGTCCAGG CAGGTAGATG ACGACCATCA
2251  GGGACAGCTT CAAGGATCGC TCGCGGCTCT TACCAGCCTA ACTTCGATCA
2301  CTGGACCGCT GATCGTCACG GCGATTTATG CCGCCTCGGC GAGCACATGG
2351  AACGGGTTGG CATGGATTGT AGGCGCCGCC CTATACCTTG TCTGCCTCCC
2401  CGCGTTGCGT CGCGGTGCAT GGAGCCGGGC CACCTCGACC TGAATGGAAG
2451  CCGGCGGCAC CTCGCTAACG GATTCACCAC TCCAAGAATT GGAGCCAATC
2501  AATTCTTGCG GAGAACTGTG AATGCGCAAA CCAACCCTTG GCAGAACATA
2551  TCCATCGCGT CCGCCATCTC CAGCAGCCGC ACGCGGCGCA TCTCGGGCAG
2601  CGTTGGGTCC TGGCCACGGG TGCGCATGAT CGTGCTCCTG TCGTTGAGGA
2651  CCCGGCTAGG CTGGCGGGGT TGCCTTACTG GTTAGCAGAA TGAATCACCG
2701  ATACGCGAGC GAACGTGAAG CGACTGCTGC TGCAAAACGT CTGCGACCTG
```

2751 AGCAACAACA TGAATGGTCT TCGGTTTCCG TGTTTCGTAA AGTCTGGAAA

2801 CGCGGAAGTC CCCTACGTGC TGCTGAAGTT GCCCGCAACA GAGAGTGGAA

2851 CCAACCGGTG ATACCACGAT ACTATGACTG AGAGTCAACG CCATGAGCGG

2901 CCTCATTTCT TATTCTGAGT TACAACAGTC CGCACCGCTG TCCGGTAGCT

2951 CCTTCCGGTG GGCGCGGGGC ATGACTATCG TCGCCGCACT TATGACTGTC

3001 TTCTTTATCA TGCAACTCGT AGGACAGGTG CCGGCAGCGC CCAACAGTCC

3051 CCCGGCCACG GGGCCTGCCA CCATACCCAC GCCGAAACAA GCGCCCTGCA

3101 CCATTATGTT CCGGATCTGC ATCGCAGGAT GCTGCTGGCT ACCCTGTGGA

3151 ACACCTACAT CTGTATTAAC GAAGCGCTAA CCGTTTTTAT CAGGCTCTGG

3201 GAGGCAGAAT AAATGATCAT ATCGTCAATT ATTACCTCCA CGGGGAGAGC

3251 CTGAGCAAAC TGGCCTCAGG CATTTGAGAA GCACACGGTC ACACTGCTTC

3301 CGGTAGTCAA TAAACCGGTA AACCAGCAAT AGACATAAGC GGCTATTTAA

3351 CGACCCTGCC CTGAACCGAC GACCGGGTCG AATTTGCTTT CGAATTTCTG

3401 CCATTCATCC GCTTATTATC ACTTATTCAG GCGTAGCACC AGGCGTTTAA

3451 GGGCACCAAT AACTGCCTTA AAAAAATTAC GCCCCGCCCT GCCACTCATC

3501 GCAGTACTGT TGTAATTCAT TAAGCATTCT GCCGACATGG AAGCCATCAC

3551 AGACGGCATG ATGAACCTGA ATCGCCAGCG GCATCAGCAC CTTGTCGCCT

3601 TGCGTATAAT ATTTGCCCAT GGTGAAAACG GGGGCGAAGA AGTTGTCCAT

3651 ATTGGCCACG TTTAAATCAA AACTGGTGAA ACTCACCCAG GGATTGGCTG

3701 AGACGAAAAA CATATTCTCA ATAAACCCTT TAGGGAAATA GGCCAGGTTT

3751 TCACCGTAAC ACGCCACATC TTGCGAATAT ATGTGTAGAA ACTGCCGGAA

3801 ATCGTCGTGG TATTCACTCC AGAGCGATGA AAACGTTTCA GTTTGCTCAT

3851 GGAAAACGGT GTAACAAGGG TGAACACTAT CCCATATCAC CAGCTCACCG

3901 TCTTTCATTG CCATACGGAA TTCCGGATGA GCATTCATCA GGCGGGCAAG

3951 AATGTGAATA AAGGCCGGAT AAAACTTGTG CTTATTTTTC TTTACGGTCT

4001 TTAAAAAGGC CGTAATATCC AGCTGAACGG TCTGGTTATA GGTACATTGA

4051 GCAACTGACT GAAATGCCTC AAAATGTTCT TTACGATGCC ATTGGGATAT

4101 ATCAACGGTG GTATATCCAG TGATTTTTTT CTCCATTTTA GCTTCCTTAG

Chloramphenicol-acetyltransferase (complementary)

4151 CTCCTGAAAA TCTCGATAAC TCAAAAAATA CGCCCGGTAG TGATCTTATT

4201 TCATTATGGT GAAAGTTGGA ACCTCTTACG TGCCGATCAA CGTCTCATTT

4251 TCGCCAAAAG TTGGCCCAGG GCTTCCCGGT ATCAACAGGG ACACCAGGAT

4301 TTATTTATTC TGCGAAGTGA TCTTCCGTCA CAGGTATTTA TTCGGCGCAA

4351 AGTGCGTCGG GTGATGCTGC CAACTTACTG ATTTAGTGTA TGATGGTGTT

4401 TTTGAGGTGC TCCAGTGGCT TCTGTTTCTA TCAGCTGTCC CTCCTGTTCA

4451 GCTACTGACG GGGTGGTGCG TAACGGCAAA AGCACCGCCG GACATCAGCG

p15A_origin

4501 CTAGCGGAGT GTATACTGGC TTACTATGTT GGCACTGATG AGGGTGTCAG
4551 TGAAGTGCTT CATGTGGCAG GAGAAAAAAG GCTGCACCGG TGCGTCAGCA
4601 GAATATGTGA TACAGGATAT ATTCCGCTTC CTCGCTCACT GACTCGCTAC
4651 GCTCGGTCGT TCGACTGCGG CGAGCGGAAA TGGCTTACGA ACGGGGCGGA
4701 GATTTCCTGG AAGATGCCAG GAAGATACTT AACAGGGAAG TGAGAGGGCC
4751 GCGGCAAAGC CGTTTTTCCA TAGGCTCCGC CCCCCTGACA AGCATCACGA
4801 AATCTGACGC TCAAATCAGT GGTGGCGAAA CCCGACAGGA CTATAAAGAT
4851 ACCAGGCGTT TCCCCCTGGC GGCTCCCTCG TGCGCTCTCC TGTTCCTGCC
4901 TTTCGGTTTA CCGGTGTCAT TCCGCTGTTA TGGCCGCGTT TGTCTCATTC
4951 CACGCCTGAC ACTCAGTTCC GGGTAGGCAG TTCGCTCCAA GCTGGACTGT
5001 ATGCACGAAC CCCCCGTTCA GTCCGACCGC TGCGCCTTAT CCGGTAACTA
5051 TCGTCTTGAG TCCAACCCGG AAAGACATGC AAAAGCACCA CTGGCAGCAG
5101 CCACTGGTAA TTGATTTAGA GGAGTTAGTC TTGAAGTCAT GCGCCGGTTA
5151 AGGCTAAACT GAAAGGACAA GTTTTGGTGA CTGCGCTCCT CCAAGCCAGT
5201 TACCTCGGTT CAAAGAGTTG GTAGCTCAGA GAACCTTCGA AAAACCGCCC
5251 TGCAAGGCGG TTTTTTCGTT TTCAGAGCAA GAGATTACGC GCAGACCAAA
5301 ACGATCTCAA GAAGATCATC TTATTAATCA GATAAAATAT TTCTAGATTT
5351 CAGTGCAATT TATCTCTTCA AATGTAGCAC CTGAAGTCAG CCCCATACGA
5401 TATAAGTTGT AATTCTCATG TTTGACAGCT TATCATCGAT AAGCT

//
