# Supplementary material for: An engineered bacterium auxotrophic for an unnatural amino acid: a novel biological containment system
Source: PeerJ. 2015 Sep 15;3:e1247. doi: 10.7717/peerj.1247 (PMC4579030; doi:10.7717/peerj.1247)
Supplement: Table S1 [file peerj-03-1247-s005.docx]

**Table S1. List of PCR primers**

Name

Sequence

immE3-1amb-s

5’-ATGTAGGGACTTAAATTGGATTTAACTTGGTTTGA-3’*

inv-immE3-as

5’-AACTTCCTCTCAAAGATATTTCTTGATA-3’

amb-immE3-confirm-s

5’-TCTTTGAGAGGAAGTTATGTAG-3’*

immE3-confirm-as

5’-ACAAAATACTCATTATCGGA-3’

*, The inserted amber stop codon is underlined.
